# Case Report: Olanzapine-Induced Severe Pancreatitis, Hyperglycemia, and Hypertriglyceridemia in a Young Female—A Rare but Life-Threatening Complication

**DOI:** 10.51894/001c.144618

**Published:** 2025-09-30

**Authors:** Sally I. Othman

**Affiliations:** 1 Department of Internal medicine Garden City Hospital

## 108

### INTRODUCTION

Olanzapine, a second-generation antipsychotic widely prescribed for bipolar disorder and schizophrenia, has been associated with rare but serious adverse effects, including pancreatitis, hyperglycemia, and hypertriglyceridemia. Drug-induced pancreatitis accounts for approximately 0.1–2% of all acute pancreatitis cases(1). Among psychotropic medications, Olanzapine has been implicated in several case reports, though its exact incidence remains unknown. This case underscores the importance of recognizing these potentially life-threatening complications of Olanzapine.

### CASE REPORT

A 29-year-old female with a history of bipolar disorder and schizophrenia presented to the emergency department (ED) with altered mental status. Her family reported a two-week history of lethargy, abdominal pain, polyuria, and polydipsia. Initially evaluated at an urgent care center, she was referred to the ED due to worsening shortness of breath (oxygen saturation 88% on room air). She required 15L of oxygen via a non-rebreather mask. Shortly after arrival, she experienced a generalized seizure that terminated spontaneously. Due to altered mentation, she was intubated for airway protection.

Laboratory evaluation revealed severe hyperglycemia (blood glucose >1400 mg/dL), acute pancreatitis (lipase 1065 U/L), and hypertriglyceridemia (triglycerides 1154 mg/dL). The patient had no prior history of diabetes, alcohol use, gallstones, or trauma. After ruling out other etiologies, including gallstones and alcohol use, Olanzapine was identified as the likely cause.

Her hospital course was complicated by prolonged ICU admission, acute kidney injury requiring dialysis, and a pulmonary embolism secondary to heparin-induced thrombocytopenia (HIT). She subsequently required a tracheostomy and PEG tube placement due to prolonged mechanical ventilation. Despite these severe complications, her condition gradually improved. She was discharged to a long-term assisted living facility. At follow-up, her kidney function fully recovered, and her tracheostomy and PEG tube were successfully removed.

### CONCLUSION

Olanzapine-induced pancreatitis, hyperglycemia, and hypertriglyceridemia, although rare, can result in life-threatening complications(2,3). Physicians should monitor patients on Olanzapine closely with periodic laboratory evaluations, including lipid panels and comprehensive metabolic profiles (CMP). Early recognition of vague or nonspecific symptoms, such as abdominal pain, lethargy, polyuria, and polydipsia, is critical to differentiating drug-induced complications from the underlying mental illness. Proactive monitoring and timely intervention can prevent severe outcomes and improve patient prognosis.

